# BT-CNN: a balanced binary tree architecture for classification of brain tumour using MRI imaging

**DOI:** 10.3389/fphys.2024.1349111

**Published:** 2024-04-11

**Authors:** Sohamkumar Chauhan, Ramalingaswamy Cheruku, Damodar Reddy Edla, Lavanya Kampa, Soumya Ranjan Nayak, Jayant Giri, Saurav Mallik, Srinivas Aluvala, Vijayasree Boddu, Hong Qin

**Affiliations:** ^1^ Department of CSE, National Institute of Technology Goa, Ponda, Goa, India; ^2^ Department of CSE, National Institute of Technology Warangal, Hanumkonda, Telangana, India; ^3^ Department of CSE, University College of Sciences, Acharya Nagarjuna University, Guntur, Andra Pradesh, India; ^4^ School of Computer Engineering, KIIT Deemed to be University, Bhubaneswar, Odisha, India; ^5^ Department of Mechanical Engineering, Yeshwantrao Chavan College of Engineering, Nagpur, India; ^6^ Department of Environmental Health, Harvard T H Chan School of Public Health, Boston, MA, United States; ^7^ Department of Computer Science and Artificial Intelligence, SR University, Warangal, Telangana, India; ^8^ Department of ECE, National Institute of Technology Warangal, Hanumkonda, Telangana, India; ^9^ Department of Computer Science and Engineering, University of Tennessee at Chattanooga, Chattanooga, TN, United States

**Keywords:** brain tumor classification, computer diagnosis, artificial intelligence, deep learning, computer-aided diagnosis, balanced binary tree

## Abstract

Deep learning is a very important technique in clinical diagnosis and therapy in the present world. Convolutional Neural Network (CNN) is a recent development in deep learning that is used in computer vision. Our medical investigation focuses on the identification of brain tumour. To improve the brain tumour classification performance a Balanced binary Tree CNN (BT-CNN) which is framed in a binary tree-like structure is proposed. It has a two distinct modules-the convolution and the depthwise separable convolution group. The usage of convolution group achieves lower time and higher memory, while the opposite is true for the depthwise separable convolution group. This balanced binarty tree inspired CNN balances both the groups to achieve maximum performance in terms of time and space. The proposed model along with state-of-the-art models like CNN-KNN and models proposed by Musallam et al., Saikat et al., and Amin et al. are experimented on public datasets. Before we feed the data into model the images are pre-processed using CLAHE, denoising, cropping, and scaling. The pre-processed dataset is partitioned into training and testing datasets as per 5 fold cross validation. The proposed model is trained and compared its perforarmance with state-of-the-art models like CNN-KNN and models proposed by Musallam et al., Saikat et al., and Amin et al. The proposed model reported average training accuracy of 99.61% compared to other models. The proposed model achieved 96.06% test accuracy where as other models achieved 68.86%, 85.8%, 86.88%, and 90.41% respectively. Further, the proposed model obtained lowest standard deviation on training and test accuracies across all folds, making it invariable to dataset.

## 1 Introduction

A Brain Tumor (BT) is an accumulation of cell abnormalities that form in the brain (Brain Tumor, 2021). The brain is protected by the skull. It will result in significant problems if there is any expansion in that restricted space. Brain tumours in general, are categorised in two types—benign and malignant. In case of benign, the cancerous cells are localised in a region and are less fatal, while the malignant tumour is fatal and has a possibility of spreading in other regions as well. The pressure inside the skull increases if such a kind of tumours grow. This will damage the brain permanently and perhaps even cause death. In UK itself, number of people diagnosed with brain tumour annually is 16,000, where only 12% of the patients survive beyond 5 years after getting diagnosed with brain tumour (BRAIN TUMOUR RESEARCH, 2024). Thus, cutting-edge techniques and procedures for screening brain cancer have been continuously developed by scientists and researchers. Magnetic Resonance Imaging (MRI) is preferred by clinical specialists over Computed Tomography (CT), despite the fact that both are commonly employed to check for anomalies in the size, shape, or placement of brain areas that help in the screening of malignancies. Consequently, MRI has been the focus of scientists and researchers. Clinicians frequently utilise traditional examination to spot brain tumours on MRI images. Clinicians are increasingly using techniques for computer—aided diagnosis, in instance, to help them diagnose brain tumours.

Gliomas are tumours of the brain that develop in glial cells. The glial cells are the brain and spinal cord’s sustaining cells. Gliomas are classified into several categories, astrocytoma being the most prevalent kind. Tumors that begin in the astrocytes are known as astrocytoma or glioblastoma. Oligodendrogliomas are tumours that begin in the oligodendrocytes. Ependymomas are tumours that begin in the ependymal cells (Cancer Research, 2024).

Meningiomas are cancers that grow from the membrane that is around the spinal cord and brain (the “meninges”). They are the most prevalent kind of adult primary brain tumour. The majority of meningioma tumours (85%–90%) are benign, with the remaining 10%–15% being atypical or malignant meningioma (cancerous). A benign meningioma brain tumour may impinge on important nerves or compress the brain, causing impairment, depending on its location and growing pace. They might potentially be life-threatening. Meningiomas are most frequent in adults aged 40 to 70, particularly in women. They are prevalent in around 3% of adults over the age of 60. Prior radiation exposure, chronic hormone usage, and genetically inherited disorders such as neurofibromatosis type 2 are the few recognised risk factors (Brigham and Women, 2024).

A development inside the pituitary gland is known as a pituitary tumour. A small gland in the brain is called the pituitary. It is located in the nasal cavity’s back. It generates hormones that have an impact on many different organs, glands, and bodily functions. Most pituitary tumours are not carcinogenic (benign). They do not circulate all around a human body. However, they have the potential to make the pituitary gland secrete hormones in inappropriate amounts, which would cause problems for the body. High secretion of hormones from pituitary tumours cause other glands to create more hormones. This will cause symptoms linked to each of the several hormones. Numerous pituitary tumours also will press on the surrounding optic nerves. Vision problems may result from this (Conditions and Diseases, 2024).

Medical imaging, sometimes referred to as radiography, is the practice of reconstructing different pictures of human organs. Medical imaging therapies employ non-invasive diagnostics to assist professionals in accurately diagnosing injuries and diseases without being intrusive. Technologies related to radiography, including CT scans and X-rays, is used in medical imaging. Nuclear Magnetic Resonance (NMR) technology is incredibly safe and produces no ionising radiation when used in MRIs. One of the safest forms of medical imaging is ultrasound imaging, which produces images through ultrasonic vibrations. The use of surface-mounted sensors to monitor electrical activity, as in electrocardiography (ECG) and electroencephalography (EEG), is a widespread method of medical imaging. However, both systems provide a change over time graph in place of a graphical representation. Using AI technology, we can improve the ability to analyse and assess data in a variety of medical imaging devices. The use of computer vision enables the detection of anomalies that the naked eye has yet to detect (TechTarget Network, 2024). Machine Learning (ML) methods can forecast the class label of unknown data items based on training data samples. ML algorithms are frequently employed in health informatics (Aishwarja et al., 2020; Khan et al., 2020; Islam et al., 2021), determining shear strength (Rahman et al., 2021), analysing consumer experience in games (Zaki et al., 2021) and predicting pandemics (Islam and Islam, 2020).

The focus of Deep Learning (DL), a branch of machine learning, is on learning data representations and hierarchical features. For feature extraction, DL techniques use a configuration of a number of layers of nonlinear processing algorithms. As we go further into the network, data abstraction is aided by the fact that each layer’s output becomes its input. A subset of deep learning (DL), called CNNs are employed to analyse visual data and are designed to need the least amount of preparation possible (LeCun, 2015). It is used to handle data in various arrays (LeCun et al., 2015) and is based on biological processes in the human brain (Matsugu et al., 2003). Feature learning and maximum accuracy, which may be attained by maximising training samples, are two advantages that CNNs have over standard machine learning and plain neural networks, leading to a more reliable and accurate model (Litjens et al., 2017). In the CNN architecture, convolutional filters serve as feature extractors, extracting more unique patterns as we go deeply (structural and spatial information). Feature extraction takes place when small filters are paired with input patterns. This is followed by the selection of the most distinctive features and the beginning of the classifier network’s training phase (LeCun et al., 2015).

The paper is organised as follows: First section discusses about brain tumour and machine learning. Section 2 elaborates on the work done earlier on the same domain and problem. The third section describes a convolutional neural network. The fourth section describes the model we propose. The fifth section elaborates on the experiments carried out with the last section concluding the paper.

## 2 Related work

Saikat Islam Khan et al. (2022) proposed two deep learning models to classify different types of brain tumors (meningioma, glioma, and pituitary) as well as binary classification of normal and abnormal cases. They utilized two publicly available datasets consisting of 152 and 3064 MRI images. In their study, the first dataset was trained using a CNN with 23 layers. However, when applied to the smaller second dataset, their “23-layer CNN” architecture encountered over fitting issues. To address this, the researchers combined transfer learning with the VGG-16 architecture and 23 layers CNN approach. Based on their experimental findings, the models achieved classification accuracies of up to 97.8% and 100% for these two datasets, respectively.

Musallam et al. (2022) proposed a preprocessing strategy for enhancing MRI image quality and a CNN architecture for the successful diagnosis of glioma, meningioma, and pituitary tumours. The model employed batch normalisation to allow faster training and simplify the activation of layer weights. The design aimed to achieve a computationally efficient model with minimal max-pooling layers, convolutional layers, and training epochs. The proposed model achieved an impressive overall accuracy of 98.22% on a dataset which has 3394 MRI images, and obatained accuracy rates of 99% for glioma, 99.13% for meningioma, and 97.3% for pituitary tumour detection, and a 97.14% accuracy rate for recognizing images without tumours.

Amin et al. (2020) proposed a fusion strategy to identify brain tumors by utilizing texture and structural data obtained from four MRI sequences: T2, Flair, T1, and T1C. Their approach involved employing a Daubechies Wavelet Kernel and Discrete Wavelet Transform (DWT) for the fusion process, which enhanced the informativeness of the tumor region. To remove noise, a Partial Differential Diffusion Filter (PDDF) was applied after the fusion. After separating the tumorous regions using a global thresholding method, the authors employed a CNN model to distinguish between the tumor and healthy regions. The proposed method was tested on five publicly available BRATS datasets.

Sultan et al. (2019) proposed a DL model built on a CNN for categorizing various forms of brain cancers using two publicly available datasets. The first dataset has 233 patients along with 3064 T1-weighted contrast-enhanced pictures, whereas the second contains 73 patients with 516 images. The model comprises two classifications: one for tumor categories (pituitary, meningioma, and glioma) and one for glioma subtypes (Grade IV, Grade III, and Grade II). For the two datasets, the proposed network topology had the greatest accuracy scores of 96.13% and 98.7% respectively.

In order to categorise brain tumour pictures without involving humans, a variety of hybrid as well as traditional machine learning models were developed and carefully evaluated. We also looked at 16 alternative transfer learning models to determine the best one for neural network-based brain tumor identification. A stacked classifier that surpasses all previously reported models was eventually suggested. It employs several cutting-edge technologies. The proposed VGG-SCNet (Majib et al., 2021) obtained an accuracy of 99.2%, recall of 99.1% and the F1-score of 99.2%.

Rehman et al. (2021) proposed a novel deep learning-based method for identifying various tumor types and detecting small brain tumors. Their approach involved utilizing a 3D CNN model to retrieve brain tumors. The obtained tumor data was then fed into a pre-trained model for feature extraction. The most relevant features were selected using the correlation-based selection technique applied to the collected attributes. To validate the selected features for the final categorization, a feed-forward neural network was employed. The method was tested and evaluated using three BraTS datasets from 2018, 2017, and 2015. The achieved accuracy for the respective datasets was reported as 98.32%, 96.97%, and 92.67% respectively.

Mishra and Verma (2022) proposed a novel attention-based photo classification paradigm for brain tumor categorization. They used a GATE-CNN model and adjusted the CNN training hyperparameters using the Adamax optimizer. The model was compared to other CNN models and evaluated on three datasets with different types of brain tumor images. The proposed model achieved higher accuracy scores than state-of-the-art CNN models, with scores of 98.27%, 99.83%, and 98.78% for the three datasets, respectively.

## 3 Convolutional neural network

This section describes a CNN and its components. A typical CNN consists of the following.

### 3.1 Convolution layer

The convolution layer is the basic component of every CNN, thus its name. This layer contains kernels, which are often a square matrix whose values are learnt by the model. This layer employs the convolution technique, which is distinct from matrix multiplication. The function is defined as follows (Ouchicha et al., 2020):

$$F\left(i,j\right)=\left(I*K\right)\left(i,j\right)=\Sigma_{m}\Sigma_{n}I\left(i+m,j+n\right)K\left(m,n\right)$$

I = image, K = 2D filter and F = feature map. Dimension of K is m*n.

There are certain characteristics that are similar across the whole dataset, or at least a large percentage of it. These characteristics are found locally in pictures and play an important role in image classification. This layer detects these features, and the output created as a result of this computation is the feature map. Nonlinearity is created by feeding the output of each convolutional layer into an activation function.

### 3.2 Batch normalisation

Batch Normalisation (BN) greatly enhances convergence during training. It entails averaging and normalising the network layer output variance (Ouchicha et al., 2020). We are given a mini-batch B = {*t*_1_, *t*_2_, …, *t*_*m*_} of size m, the normalised values $\hat{t_{1}},\hat{t_{2}},\ldots ,\hat{t_{m}}$ and corresponding linear transformations *T*_1_, *T*_2_, …, *T*_*m*_. The transformation *BN*_*γ*,*β*_: *t*_1_, *t*_2_, …, *t*_*m*_ → *T*_1_, *T*_2_, …, *T*_*m*_ is referred to as batch normalisation and is calculated as

$$\mu_{B}=\frac{1}{m}\overset{m}{\underset{i=1}{\sum }}t_{i}$$

$$\sigma_{B}^{2}=\frac{1}{m}\overset{m}{\underset{i=1}{\sum }}\left(t_{i}-\mu_{B}\right)$$

$$\hat{t_{i}}=\frac{t_{i}-\mu_{B}}{\sqrt{\sigma_{B}^{2}+\epsilon }}$$

$$T_{i}=\beta +\gamma \hat{t_{i}}\equiv BN_{\gamma ,\beta }\left(t_{i}\right)$$

### 3.3 Pooling layer

The features of the convolution layers are steadily reduced in size while the most important data is retained in the pooling layer. This layer reduces the amount of variables and calculations in the network. We define the pooling procedure by a window of size *w*_*p*_ * *w*_*p*_ that moves in step *st*_*p*_ on each feature map. We typically address this utilising two fundamental ways (Ouchicha et al., 2020):

• Max-pooling: This technique involves returning the highest local value possible for each grouping window.
• Avg-pooling: This technique involves returning the mean of the local data for each grouping window.

### 3.4 Flatten layer

Flattening refers to the procedure of converting data into a single-dimensional array to be used as input for the subsequent layer. The convolutional layer output is flattened in order to create a continuous feature vector. This flattened representation is then connected to the final classification model, which is commonly known as a fully-connected layer (Towards Data Science, 2024).

### 3.5 Fully connected layer

The dense layer, also known as a Fully Connected (FC) layer, establishes connections between every neuron in the layer and every neuron in both the same layer and the preceding layer. The number of neurons in the Dense layer is typically determined by the number of categories or classes that the network aims to learn. Each neuron in the layer contributes to the overall classification decision by taking into account the information from the preceding layer. It generates a vector with K dimensions that indicates the likelihood of categorizing each collection of photos. The FC layer links the categories to the picture, and the vector displays the results of the preceding layer, with a high value indicating the object’s location in the image.

## 4 Proposed methodology

**FIGURE 1:**
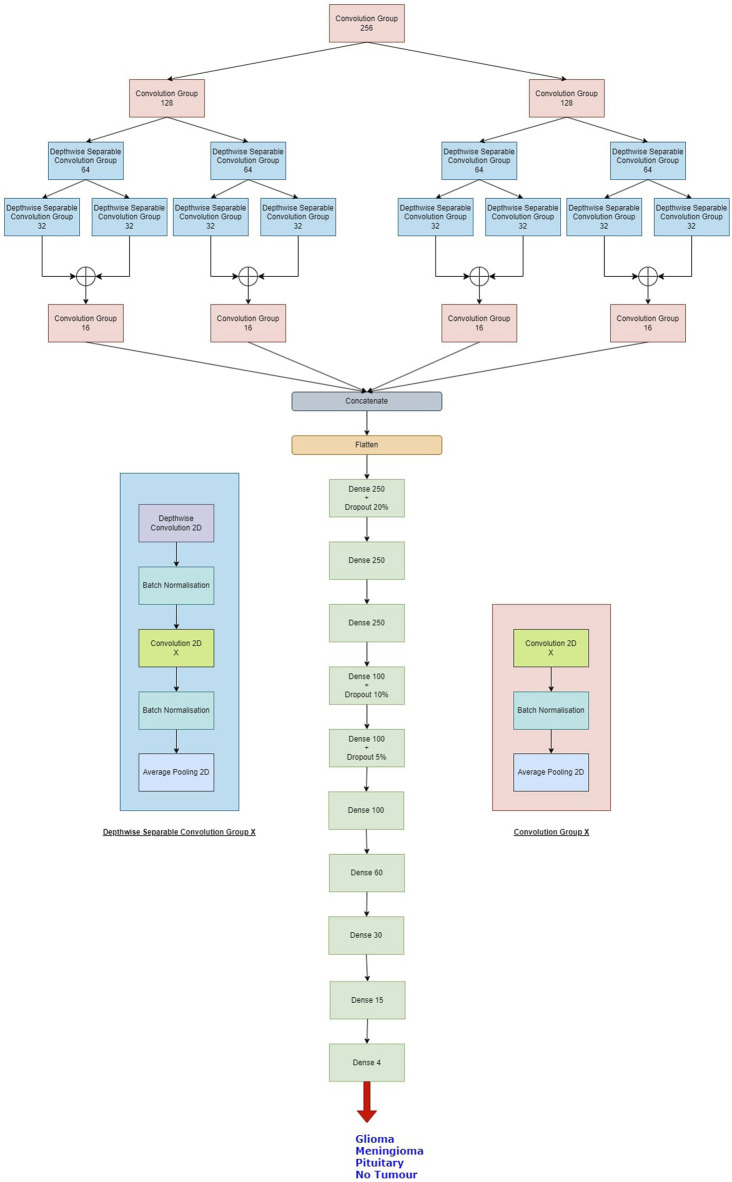
The proposed model. The depthwise separable convolution block and the convolution group block are shown on either sides of the classifier. X denotes the number of kernels used in the convolution layer.

The proposed Balanced binary Tree CNN (BT-CNN) is framed in a binary tree-like structure as shown in Figure 1. We have formed two distinct modules—the convolution group and the depthwise separable convolution group. The convolution group is formed as follows: We first start with the convolution layer with the given number of kernels, kernel size of 3*3, stride of 1*1, padding as same, activation as ReLU, with kernel inititaliser as glorot normal. This is then followed by batch normalisation. The group is ended with an average pooling layer with pool size 2*2 and stride 2*2. In the depthwise separable convolution group, the kernel size of the convolution layer is reduced to 1*1 - making it a pointwise convolution. This section is preceded with a depthwise convolution layer of kernel size 3*3 and other configurations are same as that of the convolution layer, along with a batch normalisation layer. The word “balanced” in BT-CNN stands for the optimal usage of both the groups to achieve maximum performance in terms of time and space. The usage of convolution group achieves lower time and higher memory, while the opposite is true for the depthwise separable convolution group.

Firstly broadening part of the network is described. This starts with a convolution group of 256 kernels. This gives us an output of size 100*100*256. The output of this group goes to two convolution groups of 128 kernels each. This gives us an output shape of 50*50*128 from each group. Each such group of 128 kernels passes its output to two depthwise separable convolution groups of 64 kernels, so we have a total of four such groups in this level, giving us outputs of shape 25*25*64. Each of these groups pass their outputs to two depthwise separable convolution groups of 32 kernels, so we have a total of eight such groups in this level, giving us outputs of shape 12*12*32. The total number of parameters generated in this part is 660,756.

Next shrinking part of the network is described. In this, the outputs of the sibling groups of 32 kernels are pointwise added together, forming four tensors of shape 12*12*32. The tensors are passed to individual convolution groups of 16 kernels each. This gives us an output shape of 6*6*16 from each such group. All the outputs are concatenated giving us a tensor of output shape of 6*6*64. The total number of parameters generated are 18,752.

Finally, classifier part of the network is described. In this we first flatten the output of 6*6*64 to a single tensor of 2,304. This then goes to a dense layer of 250 neurons with a dropout rate of 20%. This is again passed to two consecutive dense layers of 250 neurons each. The next three layers are dense layers with 100 neurons, with decreasing dropout rates of 10%, 5% and no dropout. We have last three layers with 60, 30 and 15 neurons each. All the layers are activated by the ReLU activation function. We then conclude the model with a dense layer of 4 neurons - one for each class, activated by the softmax activation function. The classifier generates a total of 755,469 parameters.

Overall, the model generates 1,434,957 parameters, out of which 1,430,733 parameters are trainable while 4,224 parameters are non-trainable. The same is shown in Table 1.

**TABLE 1:** Brief layered architecture of our proposed model.

Layer	Number of kernels	Output shape	Total number of parameters	Connected to
Input		200*200*1		
Convolution group	256	100*100*256	3,584	Input
Convolution group 1	128	50*50*128	295,562	Convolution group
Convolution group 2	128	50*50*128	295,562	Convolution group
Depthwise convolution group	64	25*25*64	10,304	Convolution group 1
Depthwise convolution group 1	64	25*25*64	10,304	Convolution group 1
Depthwise convolution group 2	64	25*25*64	10,304	Convolution group 2
Depthwise convolution group 3	64	25*25*64	10,304	Convolution group 2
Depthwise convolution group 4	32	12*12*32	3,104	Depthwise convolution group
Depthwise convolution group 5	32	12*12*32	3,104	Depthwise convolution group
Depthwise convolution group 6	32	12*12*32	3,104	Depthwise convolution group 1
Depthwise convolution group 7	32	12*12*32	3,104	Depthwise convolution group 1
Depthwise convolution group 8	32	12*12*32	3,104	Depthwise convolution group 2
Depthwise convolution group 9	32	12*12*32	3,104	Depthwise convolution group 2
Depthwise convolution group 10	32	12*12*32	3,104	Depthwise convolution group 3
Depthwise convolution group 11	32	12*12*32	3,104	Depthwise convolution group 3
Add		12*12*32		Depthwise convolution group 4
				Depthwise convolution group 5
Add 1		12*12*32		Depthwise convolution group 6
				Depthwise convolution group 7
Add 2		12*12*32		Depthwise convolution group 8
				Depthwise convolution group 9
Add 3		12*12*32		Depthwise convolution group 10
				Depthwise convolution group 11
Convolution group 3	16	6*6*16	4,688	Add
Convolution group 4	16	6*6*16	4,688	Add 1
Convolution group 5	16	6*6*16	4,688	Add 2
Convolution group 6	16	6*6*16	4,688	Add 3
Concatenate		6*6*64		Convolution group 3
				Convolution group 4
				Convolution group 5
				Convolution group 6
Flatten		2,304		Concatenate
Dense + Dropout	250	250	576,250	Flatten
Dense 1	250	250	62,750	Dense + Dropout
Dense 2	250	250	62,750	Dense 1
Dense 3 + Dropout 1	100	100	25,100	Dense 2
Dense 4 + Dropout 2	100	100	10,100	Dense 3 + Dropout 1
Dense 5	100	100	10,100	Dense 4 + Dropout 2
Dense 6	60	60	6,060	Dense 5
Dense 7	30	30	1830	Dense 6
Dense 8	15	15	465	Dense 7
Dense 9	4	4	64	Dense 8

## 5 Experiments

In this section, we first describe the dataset we used in the experiment. The next section describes the pre-processing applied to the images of the dataset to train the model. The third section describes the performance metrics used to evaluate the model. The fourth section describes the experiments done with the dataset. The final section is about the results obtained on performing the experiments.

### 5.1 Dataset

The Figshare Brain Tumor Dataset (Figshare, 2024), Br35H dataset (Brain Tumor Detection, 2024); Sartaj (2024) datasets are combined into single dataset (Msoud Nickparvar, 2021). There are four categories—no tumor, meningioma, pituitary and glioma—are used to categorise the dataset. The total number of images in this dataset is 7,022, and there are 1,621 images in the glioma class, 1,745 in the meningioma class, 1,757 in the pituitary class, and 2,000 in the no tumour class. The Br35H dataset did not contain any data from the tumor class.

#### 5.1.1 The figshare brain tumour dataset

This dataset includes 3,064 images which are T1-weighted contrast-enhanced from 233 individuals who had meningioma (708 slices), pituitary tumours (930 slices) and glioma (1,426 slices), which are three major forms of brain tumours.

#### 5.1.2 Dataset by Sartaj et al.

This dataset having images from 4 classes—meningioma, glioma, no tumor and pituitary tumor. The training set contains 826, 822, 827, and 395 images from respective classes. The testing set containing 100, 115, 74, and 105 images from resepetive classes.

#### 5.1.3 Br35H

This dataset contains only two classes—Yes and No, marking the presence and absence of brain tumour respectively. The dataset is balanced, both having 1,500 images.

### 5.2 Preprocessing

#### 5.2.1 Denoising using Gaussian Blur method

Denoising is the process of removing noise from an image to improve its visual quality and facilitate more accurate analysis. To decrease the noise, every image has been processed using the Gaussian function to offer the Gaussian blur feature. It is analogous to a non-uniform low-pass filter, which minimizes visual noise and irrelevant details while retaining low spatial frequency. An image is frequently convolved using a Gaussian kernel (Misra et al., 2020) to generate it. The formalisation of the Gaussian kernel is as follows:

$$G_{2D}\left(\sigma ,x,y\right)=\frac{1}{2\pi \sigma^{2}}e^{-\frac{y^{2}+x^{2}}{2\sigma^{2}}}$$

where *σ* represents the distribution’s standard deviation and x and y are the location indices. The variance of the Gaussian distribution, which defines how much blurring is present around a pixel, is determined by the amount of *σ*.

#### 5.2.2 Contrast limited adaptive histogram equalization (CLAHE)

CLAHE (Adaptive Histogram Equalization, 2024) is a variation of Adaptive Histogram Equalization (AHE) that addresses the issue of over-amplification of contrast. Unlike traditional AHE, CLAHE processes the image in small sections known as tiles rather than the entire image. It then removes the artificial boundaries between tiles by blending neighboring tiles using bi-linear interpolation. This approach can be used to enhance the contrast of an image. CLAHE can also be applied to color images. In this case, it is typically performed on the luminance channel of the image in the HSV (Hue, Saturation, Value) color space. Adjusting only the luminance channel tends to yield significantly better results compared to modifying all channels of a BGR (Blue, Green, Red) image.

#### 5.2.3 Cropping and scaling

Cropping involves removing unwanted parts of an image while retaining the region of interest. In medical imaging, cropping is often used to focus on specific anatomical structures or regions of interest within the image. It also helps standardize the input size of images, which can be important for training deep learning models that require fixed input dimensions.

In medical imaging scaling is commonly used to resize the different images while preserving its aspect ratio. This helps not only model complexity reduction but also ensures the dataset consistency.

Overall, the steps involved in the pre-processing of the images are as follows:

1. Reduction of three-channel images to one-channel, or pure grayscale images.
2. Smoothening of images using the Gausian Blur method. We keep the window size to be 3*3 with the standard deviation of 0.
3. Treatment of images to CLAHE to improve the noisy pixels. We set the clip limit to be 2 and the tile grid size to be 8*8.
4. Thresholding of images and elimination of any noise using a series of erosion and dilation operations. Here binary thresholding is used where any pixels whose grayscale value is less than 45 is set to 0.
5. Determination the image’s contours and consequent cropping the picture.
6. Resizing of the cropped images to 200*200 pixels to train and test the models.
7. One-hot encoding of the classes of the images based on the folder the image belongs to.

**FIGURE 2:**
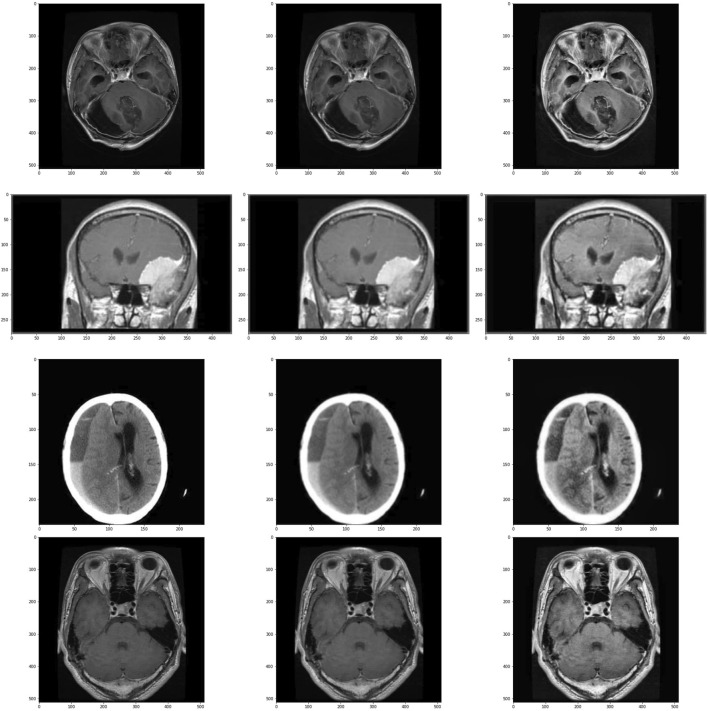
Images and its subsequent preprocessed version. Images in the left are the original images, images in center are the images after applying Gaussian Blur and on the right are the images treated with CLAHE after Gaussian Blur. First row is of glioma class, second row is of meningioma class, third row is of no tumour class and the last row is of pituitary class.

The visual output of these operations are shown in Figure 2.

### 5.3 Performance metrics

Following metrics are used to evaluate our proposed model with other models (Cheruku, 2017; Tripathi et al., 2018; Naik et al., 2023).

1. Accuracy is the fraction of predictions that our model has fulfilled, or alternatively, it is the probability that a particular input is classified correctly. Formally, it has the following definition:

$$Accuracy=\frac{number\;of\;times\;predictions\;were\;accurate}{Total\;number\;of\;times\;predicted}$$

in binary context, accuracy is defined as follows:

$$Accuracy=\frac{TN+TP}{TP+FP+TN+FN}$$

the accuracy for each class is calculated as follows:

Accuracy=NumberofcorrectlypredictedinstancesoftheclassTotalnumberofinstancesoftheclass

2. The loss function we aim to minimise is the categorical crossentropy loss. This is formally defined as follows:

$$CE=-\overset{C}{\underset{i=i}{\sum }}t_{i}log\left(s_{i}\right)$$

here, *t*_*i*_ stands for the groundtruth value and s is the vector coming out of the CNN before the loss computation, where *s*_*i*_ belongs to s. In binary context, the equation is reduced to:

$$CE=-t_{1}log\left(s_{1}\right)-\left(1-t_{1}\right)log\left(1-s_{1}\right)$$

3. Precision estimates what proportion of positive identifications are correct classified. This is helpful when the dataset is imbalanced. It is calculated as follows:

$$Precision=\frac{True\;Positive}{Total\;Predicted\;Positive}$$

4. Recall used to estimate the what proportion of actual positives are classified correctly. This is helpful when the dataset is imbalanced. It is calculated as follows:

$$Recall=\frac{True\;Positive}{Total\;Actual\;Positive}$$

5. F1-score is also useful when the dataset is imbalanced. Its a harmonic mean of both precision and recall. It is calculated as follows:

$$F1-score=2*\frac{Precision*Recall}{Precision+Recall}$$

### 5.4 Experimental setup

**FIGURE 3:**
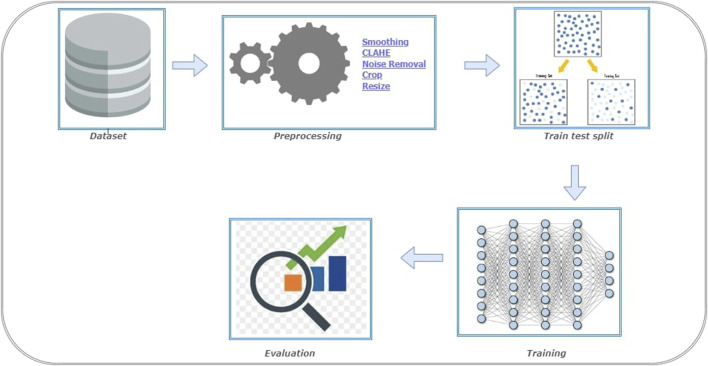
Experiment setup for Brain Tumor (BT) detection.

**FIGURE 4:**
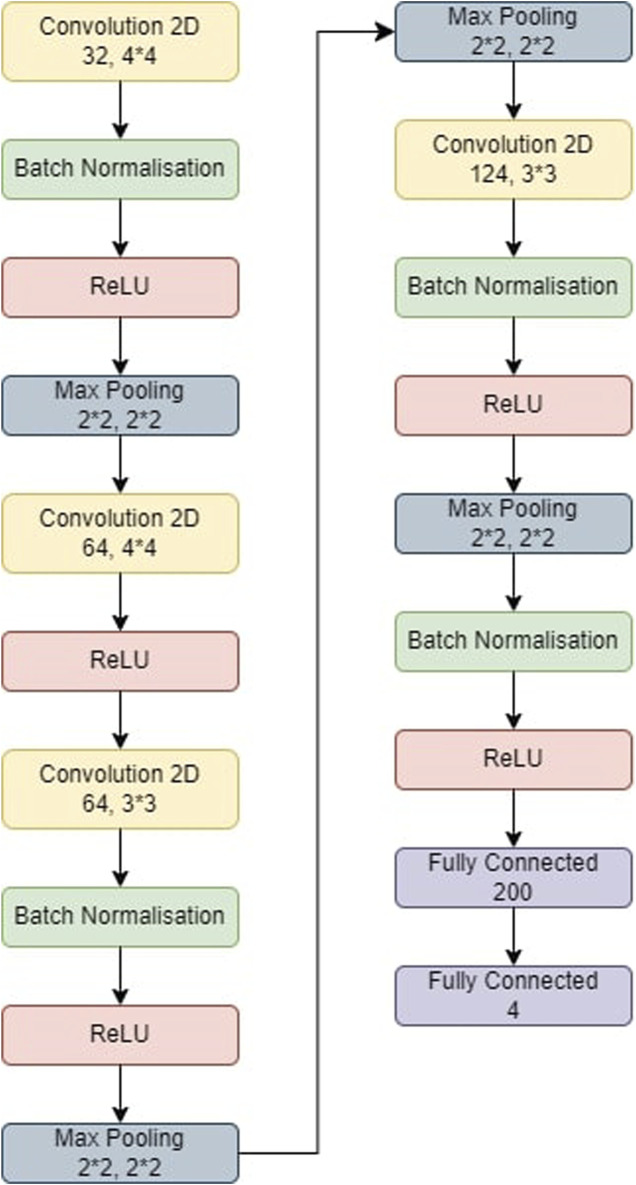
The model used for CNN-KNN. The model was first trained and then the last two fully connected layers were removed. All the images were passed through the model and we retrieved all the features of the images. All these features were trained in a KNN and the metrics were obtained.

The steps involved in BT detection procedure is explained in Figure 3. First the dataset is partitioned as per the 5-fold cross validation technique. These datasets are employed to train CNN-KNN model (Shanjida et al., 2022) (Refer Figure 4) and models proposed by Amin et al. (2020); Saikat Islam Khan et al. (2022); Musallam et al. (2022). All the programs are implemented using python and Tensorflow. The Adam optimized is used for training the proposed model. The model is trained over 20 epochs with batch size of 24. The learning rate for the optimizer was set to 0.0001, and all other properties were kept at their default settings. A categorical cross entropy is employed as loss function during the training.

The models evaluated using both training data and test data. Based on the predictions, confusion matrices are generated for each model and for each fold of the data. By analyzing the confusion matrices, we are able to assess how accurately the models classified each class.

Additionally, a classification report is obtained to gather more detailed performance metrics. The classification report provided information such as precision, recall, and F1-score. These metrics are helpful in understanding the precision (accuracy of positive predictions), recall (true positive rate), and the F1-score (a combination of precision and recall) for each class, giving us a comprehensive view of the models’ classification performance.

### 5.5 Experimental results and analysis

#### 5.5.1 CNN-KNN

The CNN-KNN model used in this experiments is shown in Figure 4. It has convolution layers, a BN layer, a max-pooling layer, a non-linear ReLU layer, and a fully linked layer. Following Batch Normalisation, ReLU, and max-pooling layers, the first convolution layer employs 44 kernels and generates 32 outputs. The second convolution layer employs 44 kernels and generates 64 outputs before being followed by a ReLU layer. Following Batch Normalisation, ReLU, and max-pooling layers, the third convolution layer employs 33 kernels and generates 84 outputs. Following BN, ReLU, and max-pooling layers, the fourth convolution layer employs 33 kernels and generates 124 outputs. Eventually, two FC layers collect features from the MRI-based pictures, which are then fed into the KNN for classification.

• In fold 1, the model obtained a training accuracy of 94.62% and a test accuracy of 90.96%. The model also classified 1,542, 1,407, 1,711, and 1,934 samples correctly for Glioma, Meningioma, Pituitary and No Tumor classes respectively. We received precisions of values 0.93, 0.94, 0.92, and 0.96; recall values of 0.95, 0.86, 0.97, and 0.97; and F1-scores of 0.94, 0.9, 0.95, and 0.96 for classes Glioma, Meningioma, Pituitary and No Tumour respectively. This results in precision, recall, and F1-score equal to 0.94 on average.
• In fold 2, the model obtained a training accuracy of 94.52% and a test accuracy of 89.75%. The model also classified 1,533, 1,377, 1,722, and 1,939 samples correctly for Glioma, Meningioma, Pituitary and No Tumor classes respectively. We received precisions of values 0.91, 0.94, 0.92, and 0.96; recall values of 0.95, 0.84, 0.97, and 0.98; and F1-scores of 0.93, 0.89, 0.95, and 0.97 for classes Glioma, Meningioma, Pituitary and No Tumour respectively. This results in precision, recall and F1-score of 0.94, 0.94, and 0.93 respectively on average.
• In fold 3, the model obtained a training accuracy of 95.25% and a test accuracy of 90.81%. The model also classified 1,554, 1,430, 1,718, and 1,925 samples correctly for Glioma, Meningioma, Pituitary and No Tumor classes respectively. We received precisions of values 0.93, 0.94, 0.94, and 0.96; recall values of 0.96, 0.87, 0.98, and 0.96; and F1-scores of 0.94, 0.9, 0.96 and 0.96 for classes Glioma, Meningioma, Pituitary and No Tumour respectively. This results in precision, recall, and F1-score equal to 0.94 on average.
• In fold 4, the model obtained a training accuracy of 94.7% and a test accuracy of 90.24%. The model also classified 1,528, 1,402, 1,720, and 1,938 samples correctly for Glioma, Meningioma, Pituitary and No Tumor classes respectively. We received precisions of values 0.92, 0.95, 0.92, and 0.96; recall values of 0.94, 0.85, 0.98, and 0.97; and F1-scores of 0.93, 0.9, 0.95, and 0.96 for classes Glioma, Meningioma, Pituitary and No Tumour respectively. This results in precision, recall, and F1-score equal to 0.94 on average.
• In fold 5, the model obtained a training accuracy of 94.63% and a test accuracy of 90.31%. The model also classifies 1,536, 1,381, 1,728, and 1,940 samples correctly for Glioma, Meningioma, Pituitary and No Tumor classes respectively. We received precisions of values 0.93, 0.94, 0.92, and 0.96; recall values of 0.95, 0.84, 0.98, and 0.97; and F1-scores of 0.94, 0.89, 0.95, and 0.97 for classes Glioma, Meningioma, Pituitary and No Tumour respectively. This results in precision, recall, and F1-score equal to 0.94 on average.

The average training accuracy across all folds is 92.37% with a standard deviation of 5.93. Similarly, the average test accuracy is 86.88% with a standard deviation of 5.54. In terms of the number of samples classified correctly, the averages across all folds are as follows: 1,548 for Glioma, 1,316 for Meningioma, 1,628 for Pituitary, and 1917 for No Tumour. The overall average precision, recall, and F1-score obtained across all folds are 0.92, 0.91, and 0.91 respectively.

#### 5.5.2 Saikat et al.

• In fold 1, the model obtained a training accuracy of 90.09% and a test accuracy of 85.48%. The model also classifies 1,605, 1,231, 1,597, and 1,829 samples correctly for Glioma, Meningioma, Pituitary and No Tumor classes respectively. We received precisions of values 0.73, 0.94, 0.95, and 1; recall values of 0.99, 0.75, 0.91, and 0.91; and F1-scores of 0.84, 0.83, 0.93, and 0.95 for classes Glioma, Meningioma, Pituitary and No Tumour respectively. This results in precision, recall and F1-score equal to 0.91, 0.89, and 0.89 respectively on average.
• In fold 2, the model obtained a training accuracy of 96.69% and a test accuracy of 92.17%. The model also classifies 1,523, 1,598, 1,742, and 1,864 samples correctly for Glioma, Meningioma, Pituitary and No Tumor classes respectively. We received precisions of values 0.91, 0.89, 0.96, and 1; recall values of 0.94, 0.97, 0.99, and 0.93; and F1-scores of 0.96, 0.93, 0.93, and 0.96 for classes Glioma, Meningioma, Pituitary and No Tumour respectively. This results in precision, recall and F1-score of 0.96 on average.
• In fold 3, the model obtained a training accuracy of 97.65% and a test accuracy of 90.96%. The model also classifies 1,589, 1,512, 1,686, and 1,977 samples correctly for Glioma, Meningioma, Pituitary and No Tumor classes respectively. We received precisions of values 0.91, 0.96, 0.99, and 0.99; recall values of 0.98, 0.92, 0.96, and 0.99; and F1-scores of 0.95, 0.94, 0.97, and 0.99 for classes Glioma, Meningioma, Pituitary and No Tumour respectively. This results in precision, recall and F1-score of 0.96 on average.
• In fold 4, the model obtained a training accuracy of 95.69% and a test accuracy of 89.03%. The model also classifies 1,572, 1,636, 1,462, and 1,957 samples correctly for Glioma, Meningioma, Pituitary and No Tumor classes respectively. We received precisions of values 0.95, 0.84, 1, and 1; recall values of 0.97, 0.99, 0.83 and 0.98; and F1-scores of 0.96, 0.91, 0.91 and 0.99 for classes Glioma, Meningioma, Pituitary and No Tumour respectively. This results in precision, recall and F1-score equal to 0.95, 0.95, and 0.94 respectively on average.
• In fold 5, the model obtained a training accuracy of 81.74% and a test accuracy of 76.78%. The model also classifies 1,452, 605, 1,655, and 1,959 samples correctly for Glioma, Meningioma, Pituitary and No Tumor respectively. We received precisions of values 0.87, 0.93, 0.77, and 0.77; recall values of 0.9, 0.37, 0.94, and 0.98; and F1-scores of 0.88, 0.53, 0.78, and 0.86 for classes Glioma, Meningioma, Pituitary and No Tumour respectively. This results in precision, recall and F1-score equal to 0.83, 0.81, and 0.78 respectively on average.

The average training accuracy across all folds is 92.37% with a standard deviation of 5.93. Similarly, the average test accuracy is 86.88% with a standard deviation of 5.54. In terms of the number of samples classified correctly, the averages across all folds are as follows: 1,548 for Glioma, 1,316 for Meningioma, 1,628 for Pituitary, and 1917 for No Tumour. The overall average precision, recall, and F1-score obtained across all folds are 0.92, 0.91, and 0.91 respectively.

#### 5.5.3 Amin et al.

• In fold 1, the model obtained a training accuracy of 96.07% and a test accuracy of 84.06%. The model also classifies 1,411, 1,459, 1,748, and 1,960 samples correctly for Glioma, Meningioma, Pituitary and No Tumor respectively. We received precisions of values 0.95, 0.9, 0.93, and 0.95; recall values of 0.87, 0.89, 0.99 and 0.98; and F1-scores of 0.91, 0.9, 0.96, and 0.97 for classes Glioma, Meningioma, Pituitary and No Tumour respectively. This results in precision, recall and F1-score equal to 0.94 on average.
• In fold 2, the model obtained a training accuracy of 52.3% and a test accuracy of 52.1%. The model also classifies 67, 1,598, 26, and 1,979 samples correctly for Glioma, Meningioma, Pituitary and No Tumor respectively. We received precisions of values 0.92, 0.47, 0.2, and 0.58; recall values of 0.04, 0.97, 0.01, and 0.99; and F1-scores of 0.08, 0.64, 0.03, and 0.73 for classes Glioma, Meningioma, Pituitary and No Tumour respectively. This results in precision, recall and F1-score equal to 0.54, 0.52, and 0.38 respectively on average.
• In fold 3, the model obtained a training accuracy of 99.09% and a test accuracy of 86.33%. The model also classifies 1,542, 113, 1,751, and 1,983 samples correctly for Glioma, Meningioma, Pituitary and No Tumor respectively. We received precisions of values 0.98, 0.98, 0.94, and 0.97; recall values of 0.95, 0.92, 0.99, and 0.99; and F1-scores of 0.96, 0.95, 0.96, and 0.98 for classes Glioma, Meningioma, Pituitary and No Tumour respectively. This results in precision, recall and F1-score of 0.97 on average.
• In fold 4, the model obtained a training accuracy of 71.88% and a test accuracy of 66.45%. The model also classifies 1,115, 113, 1,751, and 1,993 samples correctly for Glioma, Meningioma, Pituitary and No Tumor respectively. We received precisions of values 0.8, 0.79, 0.53, and 0.91; recall values of 0.69, 0.07, 1 and 1; and F1-scores of 0.74, 0.13, 0.95, and 0.7 for classes Glioma, Meningioma, Pituitary and No Tumour respectively. This results in precision, recall and F1-score of 0.76, 0.71, and 0.64 respectively on average.
• In fold 5, the model obtained a training accuracy of 53.21% and a test accuracy of 55.34%. The model also classifies 1,617, 146, 21, and 1,983 samples correctly for Glioma, Meningioma, Pituitary and No Tumor respectively. We received precisions of values 0.55, 0.78, 0.66, and 0.51; recall values of 1, 0.09, 0.01 and 0.99; and F1-scores of 0.71, 0.16, 0.02, and 0.67 for classes Glioma, Meningioma, Pituitary and No Tumour respectively. This results in precision, recall and F1-score of 0.63, 0.54, and 0.4 respectively on average.

The average training accuracy across all folds is 74.51% with a standard deviation of 20.11. Similarly, the average test accuracy is 68.86% with a standard deviation of 14.18. In terms of the number of samples classified correctly, the averages across all folds are as follows: 1,150 for Glioma, 686 for Meningioma, 1,059 for Pituitary, and 1,982 for No Tumour. The overall average precision, recall, and F1-score obtained across all folds are 0.77, 0.74, and 0.67 respectively.

#### 5.5.4 Musallam et al.

• In fold 1, the model obtained a training accuracy of 92.95% and a test accuracy of 88.97%. The model also classifies 1,617, 1,193, 1,673, and 1,989 samples correctly for Glioma, Meningioma, Pituitary and No Tumor respectively. We received precisions of values 0.79, 0.99, 0.96, and 0.98; recall values of 1, 0.73, 0.95, and 0.99; and F1-scores of 0.88, 0.84, 0.96, and 0.99 for classes Glioma, Meningioma, Pituitary and No Tumour respectively. This results in precision, recall and F1-score of 0.93, 0.92, and 0.92 respectively on average.
• In fold 2, the model obtained a training accuracy of 97.1% and a test accuracy of 94.59%. The model also classifies 1,602, 1,478, 1,743, and 1,961 samples correctly for Glioma, Meningioma, Pituitary and No Tumor respectively. We received precisions of values 0.92, 0.98, 0.97, and 0.99; recall values of 0.99, 0.9, 0.99, and 0.98; and F1-scores of 0.95, 0.94, 0.98, and 0.99 for classes Glioma, Meningioma, Pituitary and No Tumour respectively. This results in precision, recall and F1-score of 0.97 on average.
• In fold 3, the model obtained a training accuracy of 80.37% and a test accuracy of 79.57%. The model also classifies 896, 1,546 1,191, and 2,000 samples correctly for Glioma, Meningioma, Pituitary and No Tumor respectively. We received precisions of values 0.99, 0.91, 0.99, and 0.62; recall values of 0.55, 0.94, 0.68 and 1; and F1-scores of 0.71, 0.92, 0.81, and 0.77 for classes Glioma, Meningioma, Pituitary and No Tumour respectively. This results in precision, recall and F1-score of 0.88, 0.8 and 0.8 respectively on average.
• In fold 4, the model obtained a training accuracy of 74.16% and a test accuracy of 73.29%. The model also classifies 1,618, 583, 1,118, and 1,877 samples correctly for Glioma, Meningioma, Pituitary and No Tumor respectively. We received precisions of values 0.49, 0.93, 0.97, and 0.98; recall values of 1, 0.35, 0.64 and 0.94; and F1-scores of 0.66, 0.51, 0.77, and 0.96 for classes Glioma, Meningioma, Pituitary and No Tumour respectively. This results in precision, recall and F1-score of 0.85, 0.74, and 0.74 respectively on average.
• In fold 5, the model obtained a training accuracy of 94.27% and a test accuracy of 92.59%. The model also classifies 1,360, 1,517, 1,736, and 1,984 samples correctly for Glioma, Meningioma, Pituitary and No Tumor respectively. We received precisions of values 0.99, 0.92, 0.87, and 0.99; recall values of 0.84, 0.92, 0.99, and 0.99; and F1-scores of 0.91, 0.92, 0.93, and 0.99 for classes Glioma, Meningioma, Pituitary and No Tumour respectively. This results in precision, recall and F1-score of 0.94 on average.

The average training accuracy across all folds is 87.77% with a standard deviation of 8.9. Similarly, the average test accuracy is 85.8% with a standard deviation of 8.11. In terms of the number of samples classified correctly, the averages across all folds are as follows: 1,419 for Glioma, 1,263 for Meningioma, 1,962 for Pituitary, and 1,492 for No Tumour. The overall average precision, recall, and F1-score obtained across all folds are 0.91, 0.87, and 0.87 respectively.

#### 5.5.5 Proposed model

• In fold 1, our model obtained a training accuracy of 99.66% and a test accuracy of 95.8%. It also classifies 1,604, 1,592, 1,752, and 1,997 samples correctly for Glioma, Meningioma, Pituitary and No Tumor respectively. We received precisions of values 0.97, 0.99, 0.99, and 1; recall values of 0.99, 0.97, 1, and 1; and F1-scores of 0.98, 0.98, 0.99, and 1 for classes Glioma, Meningioma, Pituitary and No Tumour respectively. This results in precision, recall and F1-score of 0.99 on average.
• In fold 2, our model obtained a training accuracy of 98.97% and a test accuracy of 95.09%. It also classifies 1,597, 1,570, 1,734, and 1,995 samples correctly for Glioma, Meningioma, Pituitary and No Tumor respectively. We received precisions of values 0.95, 0.98, 0.99, and 1; recall values of 0.95, 0.99, 0.99 and 1; and F1-scores of 0.97, 0.97, 0.99, and 1 for classes Glioma, Meningioma, Pituitary and No Tumour respectively. This results in precision, recall and F1-score of 0.98 on average.
• In fold 3, our model obtained a training accuracy of 99.95% and a test accuracy of 97.01%. It also classifies 1,609, 1,625, 1,749, and 1995 samples correctly for Glioma, Meningioma, Pituitary and No Tumor respectively. We received precisions of values 0.99, 0.99, 1, and 1; recall values of 0.99, 0.99, 1, and 1; and F1-scores of 0.99, 0.99, 1, and 1 for classes Glioma, Meningioma, Pituitary and No Tumour respectively. This results in precision, recall and F1-score of 0.99 on average.
• In fold 4, our model obtained a training accuracy of 99.73% and a test accuracy of 96.01%. It also classifies 1,590, 1,624, 1,750, and 1,988 samples correctly for Glioma, Meningioma, Pituitary and No Tumor respectively. We received precisions of values 0.99, 0.98, 0.99, and 1; recall values of 0.98, 0.99, 1, and 0.99; and F1-scores of 0.99, 0.98, 0.99, and 1 for classes Glioma, Meningioma, Pituitary and No Tumour respectively. This results in precision, recall and F1-score of 0.99 on average.
• In fold 5, our model obtained a training accuracy of 99.75% and a test accuracy of 96.37%. It also classifies 1,610, 1,604, 1,749, and 1,995 samples correctly for Glioma, Meningioma, Pituitary and No Tumor respectively. We received precisions of values 0.99, 0.99, 1, and 0.99; recall values of 0.99, 0.98, 1, and 1; and F1-scores of 0.99, 0.98, 1, and 1 for classes Glioma, Meningioma, Pituitary and No Tumour respectively. This results in precision, recall and F1-score of 0.99 on average.

The average training accuracy across all folds is 99.61% with a standard deviation of 0.375. Similarly, the average test accuracy is 96.06% with a standard deviation of 0.709. In terms of the number of samples classified correctly, the averages across all folds are as follows: 1,602 for Glioma, 1,604 for Meningioma, 1,747 for Pituitary, and 1994 for No Tumour. The overall average precision, recall, and F1-score obtained across all folds is 0.99 for each of these metrics.

**FIGURE 5:**
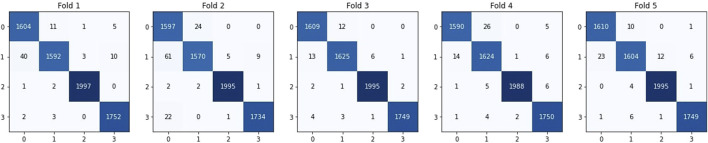
Confusion matrices derived for the proposed model. 0 represents Glioma, 1 represents Meningioma, 2 represents No Tumour and 3 represents Pituitary.

All these results are summarized in Tables 2–4. Further, the confusion matrices generated for proposed model is shown in Figure 5.

**TABLE 2:** Training and test accuracies of all frameworks across all folds.

	Training accuracy					Test accuracy				
	Musallam et al.	Saikat et al.	Amin et al.	CNN-KNN	Proposed framework	Musallam et al.	Saikat et al.	Amin et al.	CNN-KNN	Proposed framework
Fold 1	92.95	90.09	96.07	94.62	99.66	88.97	85.48	84.06	90.96	95.8
Fold 2	97.1	96.69	52.3	94.52	98.97	94.59	92.17	52.1	89.75	95.09
Fold 3	80.37	97.65	99.09	95.25	99.95	79.57	90.96	86.33	90.81	97.01
Fold 4	74.16	95.69	71.88	94.7	99.73	73.29	89.03	66.45	90.24	96.01
Fold 5	94.27	81.74	53.21	94.23	99.75	92.59	76.78	55.34	90.31	96.37

**TABLE 3:** Average Number of samples classified correctly by each Model for each Class.

	Musallam et al.	Amin et al.	Saikat et al.	CNN-KNN	Proposed framework
Glioma	1,419	1,150	1,548	1,539	1,602
Meningioma	1,263	686	1,316	1,399	1,604
Pituitary	1,492	1,059	1,628	1,720	1,747
No Tumour	1,962	1,982	1,917	1,935	1,994

**TABLE 4:** Comparison of Metrics with the proposed model.

	Musallam et al.	Amin et al.	Saikat et al.	CNN-KNN	Proposed model
*μ*(Training Accuracy) (%)	87.77	74.51	92.37	94.74	99.61
*σ*(Training Accuracy)	8.9	20.11	5.93	0.259	0.375
*μ*(Test Accuracy) (%)	85.8	68.86	86.88	90.41	96.06
*σ*(Test Accuracy)	8.11	14.18	5.54	0.432	0.709
Precision	0.91	0.77	0.92	0.94	0.99
Recall	0.87	0.74	0.91	0.94	0.99
F1-Score	0.87	0.67	0.91	0.94	0.99

## 6 Conclusion

To classify brain tumours from MRI scans a Balanced binary Tree CNN (BT-CNN) is proposed. The sample images were pre-processed by smoothing, CLAHE, denoising, cropping, and scaling to 200*200. Such pre-processed dataset is partitioned as per five fold cross validation. The proposed model along with other state-of-the-art models are trained for 20 epochs using batch size of 24. To reduce the cross-categorical entropy loss function, Adam optimizer is employed. From the experimtal resutls we observed that our proposed model outperformed the models in our study, achieving average training accuracy of 99.61% and test accuracy of 96.06%. Our model also obtained Precision, Recall and F1-score of 0.99. Our propsoed model has one of the lowest standard deviation in training and test accuracy over all folds. Future study will focus on developing a model that shows an improved performance at categorization.

## Data Availability

The original contributions presented in the study are included in the article/Supplementary material, further inquiries can be directed to the corresponding author.
